# Genome-wide alteration of 5-hydroxymenthylcytosine in a mouse model of Alzheimer’s disease

**DOI:** 10.1186/s12864-016-2731-1

**Published:** 2016-05-20

**Authors:** Liqi Shu, Wenjia Sun, Liping Li, Zihui Xu, Li Lin, Pei Xie, Hui Shen, Luoxiu Huang, Qi Xu, Peng Jin, Xuekun Li

**Affiliations:** Department of Human Genetics, Emory University School of Medicine, Atlanta, GA 30022 USA; Institute of Genetics, College of Life Sciences, Zhejiang University, 310058 Hangzhou, China; The Children’s Hospital, School of Medicine, Zhejiang University, 310052 Hangzhou, China; Institute of Translational Medicine, School of Medicine, Zhejiang University, 310029 Hangzhou, China; Department of Endocrinology, Wuhan Central Hospital, 430014 Wuhan, China; National Laboratory of Medical Molecular Biology, Institute of Basic Medical Sciences & Neuroscience Center, Chinese Academy of Medical Sciences and Peking Union Medical College, 100005 Beijing, China

**Keywords:** Alzheimer’s disease, 5-hydroxymethylcytosine, DNA demethylation, Amyloid peptide

## Abstract

**Background:**

Alzheimer’s disease (AD) is the most common form of neurodegenerative disorder that leads to a decline in cognitive function. In AD, aggregates of amyloid β peptide precede the accumulation of neurofibrillary tangles, both of which are hallmarks of the disease. The great majority (>90 %) of the AD cases are not originated from genetic defects, therefore supporting the central roles of epigenetic modifications that are acquired progressively during the life span. Strong evidences have indicated the implication of epigenetic modifications, including histone modification and DNA methylation, in AD. Recent studies revealed that 5-hydroxymethylcytosine (5hmC) is dynamically regulated during neurodevelopment and aging.

**Results:**

We show that amyloid peptide 1–42 (Aβ1-42) could significantly reduce the overall level of 5hmC in vitro. We found that the level of 5hmC displayed differential response to the pathogenesis in different brain regions, including the cortex, cerebellum, and hippocampus of APP-PSEN1 double transgenic (DTg) mice. We observed a significant decrease of overall 5hmC in hippocampus, but not in cortex and cerebellum, as the DTg mice aged. Genome-wide profiling identified differential hydroxymethylation regions (DhMRs) in DTg mice, which are highly enriched in introns, exons and intergenic regions. Gene ontology analyses indicated that DhMR-associated genes are highly enriched in multiple signaling pathways involving neuronal development/differentiation and neuronal function/survival.

**Conclusions:**

5hmC-mediated epigenetic regulation could potentially be involved in the pathogenesis of AD.

**Electronic supplementary material:**

The online version of this article (doi:10.1186/s12864-016-2731-1) contains supplementary material, which is available to authorized users.

## Background

Alzheimer’s disease (AD) is a progressive neurodegenerative disease involving multiple pathologic processes and is characterized by the deposition of amyloid beta (Aβ) peptide, neurofibrillary tangles (NFTs) composed of hyperphosphorylated protein tau, and neuronal cell death [1, 2]. Recent studies indicate that epigenetic pathways could be involved in the pathogenesis of AD [3, 4]. DNA methylation (5-methylcytosine, 5mC) plays important roles in regulating gene expression and is involved in multiple neurodevelopmental and neurodegenerative disorders [5–7].

Changes in 5mC at the global level or at specific loci are seen in the brain tissues of AD model mice, as well as AD patients [4, 8–12]. Although some regions and loci show hypermethylation [13], global DNA hypomethylation has been observed in the entorhinal cortex of some AD patients [14], suggesting DNA methylation is differentially affected in a region- and loci-specific manner. Previous studies also found that the promoter regions of amyloid precursor protein (APP) and presenilin 1 (PSEN1) displayed age-dependent hypomethylation [10, 15–17]. Furthermore, in vitro hypomethylation of PSEN1 increased the cleavage of APP and the production of Aβ in a neuroblastoma cell line [18]. Recently, two large-scale epigenome-wide association studies uncovered the alteration of site-specific methylation in the brains of AD patients [11, 12]. These results imply DNA methylation could play important roles in the pathogenesis of AD.

Recently, another cytosine modification, 5-hydroxymethylcytosine (5hmC), was identified and found to be highly abundant in the neuronal system [19–21]. Ten-eleven translocation (Tet) family proteins, including Tet1, Tet2, and Tet3, are known to catalyze the hydroxylation of 5mC to 5hmC [19, 22–24]. Recent studies strongly indicate that 5hmC not only serves as an intermediate of DNA demethylation, but can also perform as a stable epigenetic marker. 5hmC is ~10-fold more enriched in neurons than other cell types, and it is acquired globally and exhibits dynamic features and region-specific patterns during postnatal development and aging of the neuronal system [20, 25, 26]. Genome-wide studies reveal that 5hmC can be enriched in distinct genomic regions, such as gene bodies, promoters, and distal regulatory regions [27–29], and the enrichment of 5hmC ate gene bodies could be positively correlated with transcriptional level, which might be achieved via interaction with histone modifications [30–34]. The alteration of global 5hmC and differentially hydroxymethylated regions (DhMRs) are seen in several neurodevelopmental diseases, including Rett syndrome, autism, and neurodegenerative diseases like Huntington’s disease and fragile X-associated tremor/ataxia syndrome (FXTAS), suggesting 5hmC could play important roles in neurological diseases [20, 25, 35–37].

Despite the clear alteration of DNA methylation observed in AD, whether and how 5hmC is involved in AD pathogenesis still remain largely unknown. Using an immunostaining method, Condliffe et al. found a significant decrease of global 5hmC in the cortex and cerebellum of AD patients [38]. In contrast, using the same technique, other studies reported an increase of global 5hmC in both AD mouse model and patients samples [39–41]. To study the alteration of 5hmC in AD and explore the potential role(s) of 5hmC-mediated epigenetic regulation in the pathogenesis of AD, here we investigated the effect of Aβ on 5hmC in vitro and found Aβ treatment could significantly decrease the level of 5hmC in a dose-dependent pattern. Furthermore, we found that 5hmC levels displayed an age-dependent decrease in the hippocampus, but not in the cortex and cerebellum, of APP-PSEN1 double-transgenic (DTg) mice. Using a chemical-labeling 5hmC enrichment approach, we performed genome-wide profiling of 5hmC. We found that, although AD pathogenesis did not change the overall distribution of 5hmC, there were differentially hydroxymethylated regions (DhMRs) in DTg mice. The DhMRs identified are involved in a number of neuronal signaling pathways, indicating a 5hmC-mediated epigenetic pathway could potentially play important roles in the pathogenesis of AD.

## Results

### Aβ reduces the global level of 5-hydroxymethylcytosine in vitro

**Fig. 1:**
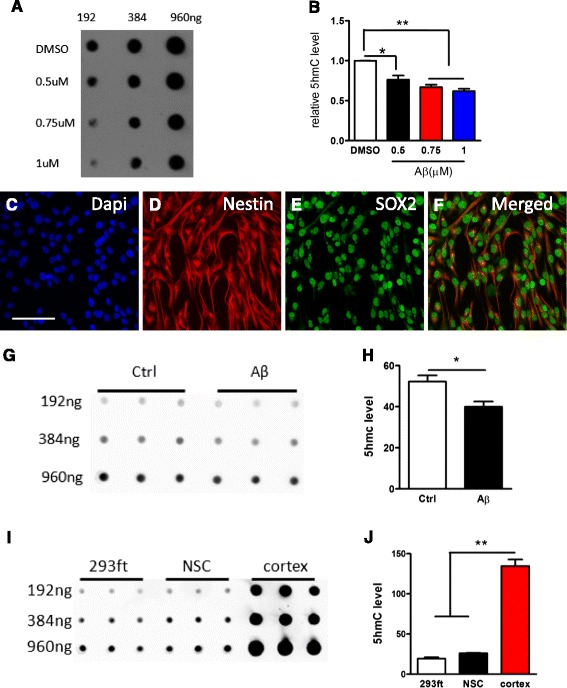
The effects of Aβ(1–42) peptide on 5hmC level in vitro. **a**-**b** Dot-blot assay shows Aβ treatment significantly decreased total 5hmC levels in a dose-dependent manner in HEK293ft cells. Aβ at 1-μM concentration is the most effective at decreasing 5hmC levels (*p < 0.05;** *p* < 0.01, unpaired *t*-test). **c**-**f** The cultured aNSCs are positive for neural stem cell markers Nestin (**d**) and SOX2 (**e**). **g** The representative images of dot-blot assay of Aβ treatment on 5hmC level in aNSCs. **h** The quantification result indicates Aβ at 1-μM concentration significantly decreases 5hmC level in aNSCs (*p < 0.05;** *p* < 0.01, unpaired *t*-test). **i**-**j** Dot-blot assay indicates that the global level of 5hmC in cortex is significantly higher than in HEK293ft cells and aNSCs (*p < 0.05;** *p* < 0.01, unpaired *t*-test)

Aβ deposition is one of the hallmarks of AD pathogenesis, and is known to induce neuronal cell death and other neuronal pathogenic outcomes. To study the roles of 5-hydroxymethylcytosine (5hmC)-mediated epigenetic modification in AD pathogenesis, we first studied the effect of Aβ(1–42), a toxic form of peptide associated with AD, on the level of 5hmC with cultured cells. After being treated with Aβ peptide for 48 h, the overall level of 5hmC in HEK293ft cells decreased, and Aβ peptide at a 1-μM concentration was the most effective dose (Fig. 1a-b). To ensure equal spotting of total DNA on the membrane, the same blot was then stained with 0.02 % methylene blue (Additional file 1: Figure S1).

Considering cognitive function is severely impaired and the roles of adult neurogenesis in learning and memory, we further tested the effect of Aβ peptide on 5hmC levels in adult neural stem cells (aNSCs). ANSCs harbors in specific regions, subventricular zone of lateral ventricle and subgranular zone of dentate gyrus of adult mammalian brain, and is involved in neurological disorders including AD. The isolated aNSCs were positive for neural stem cell markers Nestin and Sox2 (Fig. 1c-f). After treated with Aβ peptide at a 1-μM concentration for 48 h, the level of 5hmC was also significantly decreased in the cultured aNSCs (Fig. 1g-h). We also compared the overall level of 5hmC in HEK293ft cells, aNSCs and neuronal tissues. We found that 5hmC level was significantly higher in neuronal tissues than in HEK293ft and aNSCs cells, which both are capable of proliferation (Fig. i-j). Taken together, these results indicate that Aβ peptide could significantly affect the level of 5hmC in multiple cultured cells.

### 5-hydroxymethylcytosine level decreases during aging in an AD mouse model

**Fig. 2:**
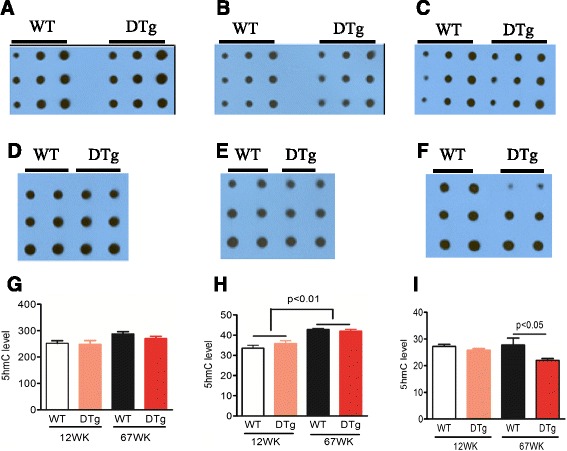
Reduced 5hmC level at specific brain regions in a mouse model of AD. **a**-**f** Representative images of 5hmC dot-blot assay of 12- and 67-week-old WT and DTg mice cortex (**a**, **d**. *n* = 3), cerebellum (**b**, **e**. *n* = 3), and hippocampus (**c**, **f**. *n* = 3). **g**-**i** The quantitative results indicated that the global levels of 5hmC did not show significant difference in cortex and cerebellum of WT and DTg mice (**g**, **h**). In hippocampus, the overall abundance of 5hmC was significantly decreased in DTg mice compared to WT mice at 67-week stage while it did not show observable difference between WT and DTg mice at 12-week stage (**i**). (ANOVA post Bonferroni’s Multiple Comparison Test, mean ± s.e.m. *p < 0.05, **p < 0.01)

Previous studies have indicated that 5hmC could be acquired in the brain during postnatal development and aging [20, 25]. To examine whether the level of 5hmC is affected during AD pathogenesis, we dissected multiple brain regions, including cortex, cerebellum, and hippocampus from 12-week-old (adult) and 67-week-old (aged) wild-type (WT) and APP-PSEN1 double transgenic (DTg) mice. Consistent with our previous study [20], from 12 weeks to 67 weeks, 5hmC exhibits no or slight acquisition in the cortex and cerebellum of both WT and DTg mice (Fig. 2b, e, h). Quantification results showed no significant difference of these brain two regions between WT and DTg mice (Fig. 2a, b, d, e, g, h). In hippocampus, there is no significant change in 5hmC in WT mice during aging; however, at the 67-week time point, the 5hmC level of DTg mice hippocampus decreased significantly compared to age-matched WT control (Fig. 2c, f, i). Collectively, these results indicate that the global level of 5hmC is affected in specific brain regions during AD pathogenesis.

### Acquisition of 5hmC on gene bodies is altered in aged AD mice

**Fig. 3:**
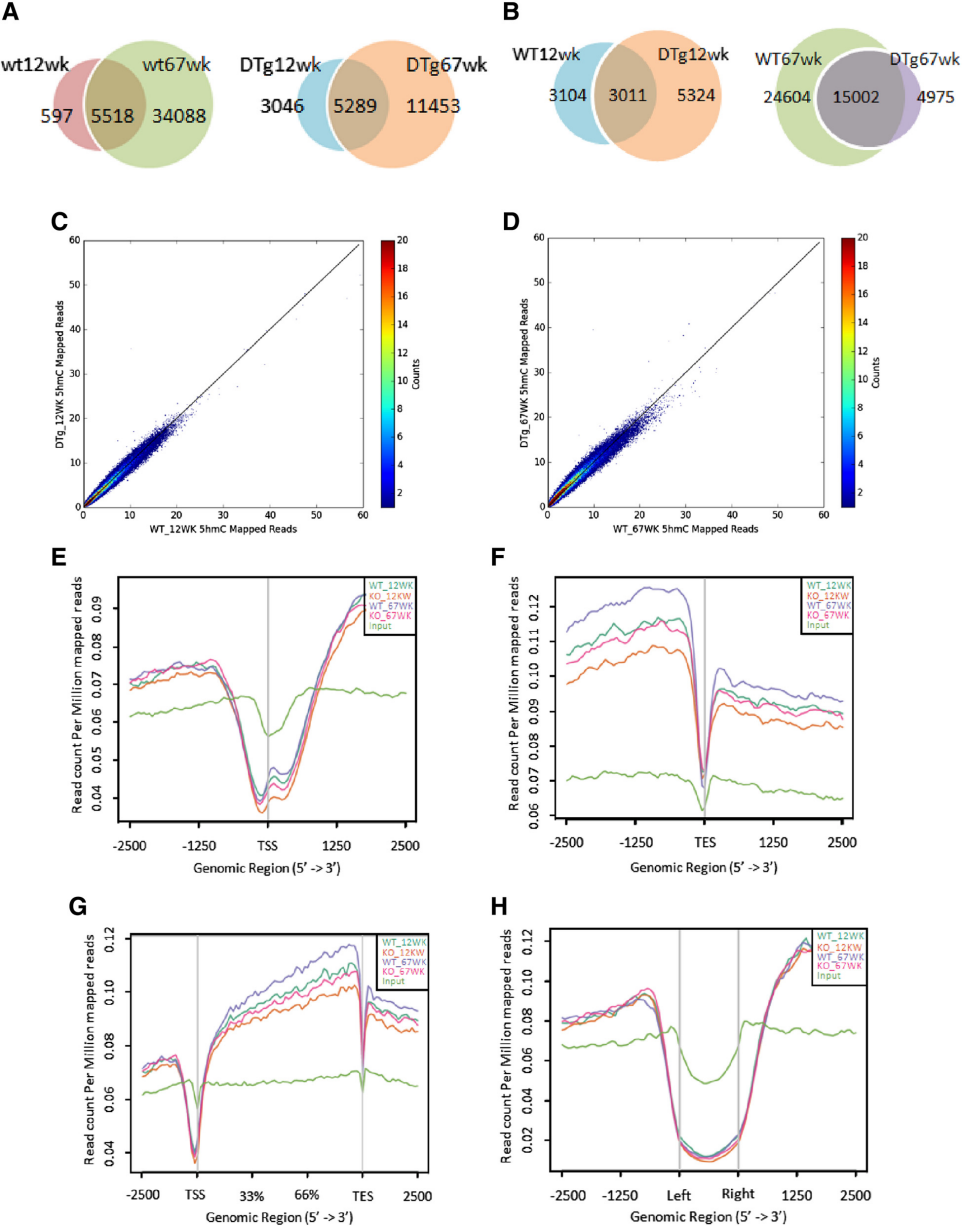
Genomic features of 5hmC peaks in hippocampus between WT and DTg mice. **a**-**b** 6115, 39,606, 8335, and 19,977 of 5hmC peaks were called from 12- and 67-week-old WT and age-matched DTg mice biological replicates, respectively. 5518 peaks overlapped between 12- and 67-week-old WT mice. 5289 peaks overlapped between 12- and 67-week-old DTg mice. 3011 peaks overlapped between 12-week-old WT and DTg mice. 15,002 peaks overlapped between 67-week-old WT and DTg mice. **c** Genome-wide 5hmC reads were counted within each 10-kb bin in WT_12 WK and DTg_12WK mice genome. 5hmC levels were not significantly different between adult WT and DTg mice. **d** Genome-wide 5hmC reads densities were higher in WT mice than in DTg mice. **e**, **f**, **g** and **h** Normalized 5hmC read densities on transcription end sites (TESs), transcription start sites (TSSs), gene bodies, and CpG islands. The enrichment of 5hmC significantly decreased in adult and aged DTg mice compared to age matched WT mice, but no significant difference was observed at TSS, TES, and CpG islands. *t*-test, *p* < 0.01

To explore whether the distribution features of 5hmC in the genome are altered during AD pathogenesis, we employed a previously established 5hmC chemical labeling and affinity purification method [26] and performed 5hmC genome-wide profiling. Based on dot-blot results, our subsequent study focused on hippocampus. To perform genome-wide sequencing of 5hmC, hippocampus tissues were dissected from three adult (12-week) DTg mice and three littermate WT mice; at the 67-week time point (aged), hippocampus tissues were dissected from two DTg mice and two WT littermate mice. Through deep-sequencing, 11–23 million total reads and around 9–18 million monoclonal reads were generated from each sample (Additional file 2: Table S1). Sequence data were analyzed using our established pipeline [20], and peaks were identified by MACS software [42]. 6115 and 8335 5hmC peaks were called from adult WT and DTg mice, respectively (Fig. 3a, b). At the 67-week time point, 39,606 and 19,977 peaks were identified from WT and DTg mice, respectively (Fig. 3a, b). Although during the aging process, AD pathogenesis did not significantly affect the shared peaks between adult and aged mice of each genotype: 5518 peaks were shared between adult and aged WT mice, and 5289 peaks were shared between adult and aged DTg mice, the number of total peaks decreased remarkably in aged DTg mice compared to WT littermates. (Fig. 3a, b). At the chromosome level, there was no visible difference between WT and DTg mice (Additional file 1: Figure S2). Consistent with our previous work [20], depletion of 5hmC on the X chromosome was also observed in both WT and DTg mice (Additional file 1: Figure S2).

We next determined the distribution of 5hmC on distinct genomic regions. Genome-wide 5hmC read density was detected in the DTg mouse model (Fig. 3c, d). At the 12-week point, we saw no significant difference between WT and DTg mice (Fig. 3c). However, until the 67-week point, the shifted entire plot pattern suggested that WT bins had more 5hmC reads (Fig. 3d). Furthermore, the distribution of 5hmC was studied at 2.5 kb up- and downstream of transcription end sites (TESs), transcription starting sites (TSSs), gene bodies, and CpG islands by ngs-plot software (Fig. 3e-h). We found that the distribution of 5hmC showed no observable differences on TESs and CpG islands between WT and DTg mice (Fig. 3e, h), whereas it was slightly increased on TSSs in WT mice compared to age-matched DTg mice (Fig. 3f). From the 12-week to 67-week time point, 5hmC was acquired on gene bodies in both WT and DTg mice (Fig. 3g), however, at 12- and 67-week time points, the enrichment of 5hmC in gene bodies showed a significant decrease in DTg mice compared to age-matched WT mice (*t*-test, *p* < 0.0001), suggesting the acquisition of 5hmC in gene bodies was inhibited during AD pathogenesis.

### Differential hydroxymethylated regions (DhMRs) associated with AD

**Fig. 4:**
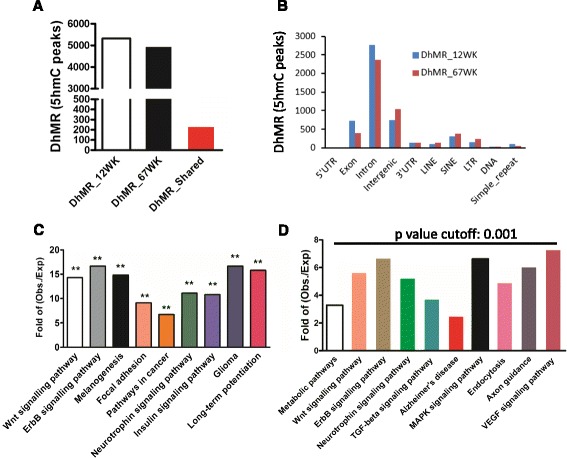
Identification and characterization of DhMRs in AD mouse model. **a** Compared to age-matched WT mice, 5324 and 4975 DhMRs were identified from adult and aged DTg-specific mice, respectively. 244 specific DhMRs were shared between adult and aged DTg mice, which did not exist either in adult or aged WT mice. **b** The distribution features of DhMRs identified in adult and aged DTg mice, respectively. DhMRs highly enrich in introns, exons and intergenic regions. Pearson’s Chi-squared test with Yates’ continuity correction was performed using their absolute mapped reads inside and outside of each genomic feature. P-values for these tests were significant (<2.2e-16). **c** KEGG assay shows DhMR-associated genes are significantly enriched in multiple neuronal signaling pathways. **d** KEGG assay indicates aged mice specific DhMRs-associated genes are also significantly enriched in multiple pathways, including Alzheimer’s disease

The partial overlapping of 5hmC peaks in WT and transgenic mice suggested differential hydroxymethylation. We next sought to identify differential hydroxymethylation regions (DhMRs) in the genome. Compared to age-matched WT mice, 5324 and 4975 specific DhMRs were identified in adult and aged DTg mice, respectively (Fig. 4a). Among them, 244 specific DhMRs were shared between adult and aged DTg mice, which did not appear in either adult or aged WT mice (Fig. 4a). The DhMRs identified in adult and aged DTg mice displayed similar distribution trend: abundantly enriching in intron, exon and intergenic regions (Fig. 4b), suggesting a high conservation during AD progress.

To further reveal the biological function of identified DhMRs in both adult and aged DTg mice, the genes associated with these DhMRs were extracted for enrichment analysis. We found 167 genes associating with the identified DTg-specific DhMRs (Additional file 3: Table S2). DhMR-associated genes were highly enriched in multiple signaling pathways, such as the Wnt and ErbB pathways, which play important roles in the neuronal system (Fig. 4c). Furthermore, we generated 4557 aged DTg mice-specific peaks, and extracted 2424 genes, which associated with those peaks (Additional file 4: Table S3). Gene Ontology assay showed that those genes significantly enriched in some pathways, such as Alzheimer’s disease pathway, Wnt signalling, etc. (Fig. 4d). Taken together, these results suggest that the specific enrichment of 5hmC could play some role(s) in regulating the expression of genes related neuronal function, and involve in the pathogenesis of Alzheimer’s disease.

**Fig. 5:**
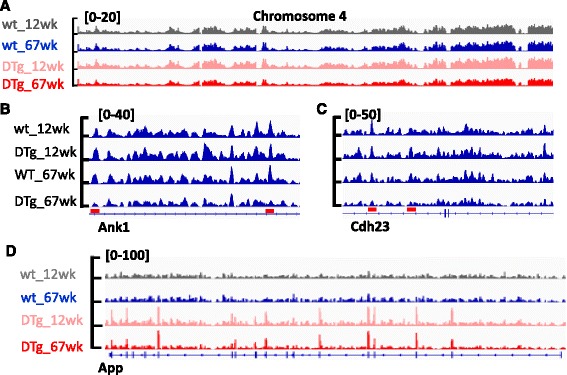
Identification and characterization of DhMRs in AD mouse model. **a** A representative IGV image shows the reduction of overall 5hmC in aged DTg mice compared to WT mice and adult DTg mice. **b**-**c** Representative IGV images show the decrease of 5hmC in some genomic regions of Ank1 and Cdh23. **d** 5hmC highly enriched in the gene body of APP gene in adult and aged DTg mice compared to WT mice. MACS software default statistic test, *p*-value cutoff: 1X10^−5^

Two recent large-scale studies identified alterations of DNA methylation in some loci of AD patients [11, 12], suggesting DNA demethylation might be involved in this process. An IGV image showed the overall reduction of 5hmC peaks in aged DTg mice compared to age matched WT mice (Fig. 5a). Interestingly, in DTg mice specific and aged DTg mice specific peaks associated genes identified in our current study, two genes are also found in the two EWAS AD studies: Ank1, Cdh23 (Fig. 5b, c). We next examined the 5hmC distribution profile of the genes identified in those two EWAS studies, and we found the enrichment of 5hmC peaks in some regions of those genes decreased (Additional file 1: Figure S3). These data suggest DNA methylation is altered in AD associated loci, and the potential roles of DNA demethylation in the pathogenesis of AD.

Previous studies also noted that the enrichment of 5hmC in gene bodies might be positively correlated with gene expression [37]. In our AD model mice, two AD-associated genes, APP and PSEN1, were over-expressed. We found 5hmC peaks highly enriched in APP gene bodies, especially in exons, in both adult and aged DTg mice compared to WT mice (Fig. 5d, over two-fold difference, p < 1x10^−5^). It is of interest to note that we did not see significant difference of 5hmC distribution in PSEN1. These data indicated that 5hmC enrichment could be one of the mechanisms promoting gene expression.

## Discussion

In the present study, we performed in vitro and in vivo studies to characterize the alterations of 5hmC-mediated DNA demethylation in a mouse model of AD. We found the overall level of 5hmC is significantly higher in brain tissues than in cell lines and adult neural stem cells. AD pathogenic protein amyloid peptide led to a decrease of global 5hmC in cultured cells. Our in vivo study also found the level of 5hmC decreased in one specific brain region, i.e., the hippocampus, but not other studied brain regions of AD mice during the pathogenesis of AD. Genome-wide profiling results indicated that the distribution of 5hmC altered in distinct genomic regions, especially in gene bodies. The differentially hydroxymethylated regions (DhMRs) identified in hippocampus of aged D-Tg mice displayed high enrichment of multiple signaling pathways that are related to neuronal development and neuronal function. Some AD-associated genes showed altered hydroxymethylation. Our study therefore uncovered new roles for 5hmC-mediated epigenetic modification in neurologic disorders and revealed a new layer of the pathogenic mechanism of AD.

Global and site-specific alterations of DNA methylation had been identified in AD [10, 14, 15, 43–45]. Epigenome-wide association with AD revealed the relationship between differential methylation of CpGs and the expression of nearby genes, some of which are connected to a known AD susceptibility network [11, 12]. Previous studies had inconsistently reported about the alteration of DNA demethylation [38–41]. In our present study, Aβ treatment led to a significant decrease in global 5hmC level of different types of cells in vitro. The significant alteration of demethyaltion was only observed in hippocampus but not in cortex and cerebellum during the ageing of AD model mice, suggesting the alteration of demethylation is region specific, which might explain the data contradiction between different studies. Considering the acquired Aβ deposition during the ageing of AD model mice, these results indicated the increased Aβ deposition decreased overall 5hmC level. Together with previous studies, it supports the idea that dysregulation of DNA demethylation is age- and region/loci specific, indicating an interaction between amyloid accumulation and DNA modification.

Previous studies have found 5hmC is highly enriched in the neuronal system [19, 26], and the enrichment is enhanced and displays dynamic features during postnatal development and aging of the brain [20, 46], indicating 5hmC could be important for brain function. Subsequent studies did find that 5hmC-mediated epigenetic modification is involved in multiple neurological disorders, including autism spectrum disorders, Huntington’s disease, and FXTAS [20, 25, 36, 37]. Moreover, the Rett syndrome protein MeCP2 could bind to 5hmC, and its dosage is negatively correlated with 5hmC level [20, 35]. Our present results revealed that the overall distribution features of 5hmC in TSSs, TESs, and CpG islands did not change, but the enrichment of 5hmC in gene bodies was significantly decreased compared with age-matched WT mice. Previous research found that the 5hmC level in gene bodies is positively correlated with gene expression [20, 47]. Our results that the highly enrichment of 5hmC in the gene body of APP further supports this concept. Considering no observable difference of 5hmC in another overexpressed gene PSEN1, it suggests that 5hmC enrichment in gene body is one but all of mechanisms to modulate gene expression. Further, our present studies identified some DhMR-associated genes enriched in multiple signaling pathways that are related to neuronal function and neurological disorders [20, 25, 36, 37]. These results suggest a potential mechanism to explain how 5hmC-mediated epigenetic modification functions.

Our present studies also uncovered altered demethylation in some specific loci in aged D-Tg mice, and some loci were related with the onset and progress of AD. Interestingly, the loci with altered DNA demethylation identified in our study were also found displaying altered DNA methylation profile by two large-scale studies in human [11, 12]. Although our present and these two studies mainly provided the relevant evidence, all these results suggest DNA methylation and demethylation changes are involved in AD. Further experimental work needs to be conducted to address the mechanism how altered DNA methylation and demethylation affect the onset and pathogenesis of AD.

## Conclusion

Overall, our results indicate that not only is the global level but also the distribution features of 5hmC altered in AD model mice. Aβ treatment decreased 5hmC both in vitro and in vivo. The acquisition of 5hmC in gene bodies during postnatal development was significantly inhibited in the hippocampus of AD model mice, although the overall features in TSSs, TESs, and CpG islands were unaffected. The DhMR-associated genes identified in AD mice are specifically related to some signaling pathways that play a role in neuronal function and neurological disorders. Taken together, our present results argue that 5hmC-mediated epigenetic modification could have an important function in AD.

## Methods

### Animals

Twelve- and 67-week-old wild-type (WT) and APP/PS1 double transgenic littermate mice were used in this study [48]. Mice were maintained at ambient temperature (22-24 °C) on a 12:12 light/dark cycle with free access to food and water. All animal procedures were performed according to protocols approved by Emory University Institutional Animal Care and Use Committee.

### Genomic DNA isolation and 5hmC dot-blot

Genomic DNA was extracted as described previously [25]. Briefly, the dissected brain samples or cells were homogenized in lysis buffer (5 mM EDTA, 0.2 % SDS, 200 mM NaCl in 100 mM Tris–HCl, pH 8.5) supplemented with proteinase K, and samples were kept at 56 °C overnight. The second day, an equal volume of phenol:chloroform:isoamyl alcohol (25:24:1, P-3803, Sigma) was added, mixed completely, and centrifuged at 14,000 rpm for 10 min. An equal volume of isopropanol was added to the supernatant to precipitate DNA, which was dissolved with 10 mM Tris–HCl (pH 8.0).

5hmC dot-blot was performed as before [20]. In brief, genomic DNA was spotted on an Amersham Hybond-N+ membrane (GE Healthcare), followed by baking at 80 °C for 30 min. The membrane was incubated with polyclonal 5hmC antibody (Active Motif, #39769) overnight at 4 °C. The second day, a horseradish-peroxidase-conjugated secondary antibody against rabbit was used to probe.

### Cell culture and Aβ treatment

HEK293 cells were maintained in DMEM supplemented with 10 % fetal bovine serum, 2 mM glutamine, and 100 U penicillin–streptomycin at 37 °C in a humidified incubator containing 5 % CO_2_. Cells were treated with Aβ peptide (Sigma, A9810) at a concentration of 0.5, 0.75, or 1 μM for 48 h, respectively. The isolation, culture and determination of adult neural stem cells were performed as described previously [49].

### Immunocytochemistry

The cultured aNSCs were fixed with 4 % parafromaldehyde for 30 min at room temperature, followed by washing with cold PBS for 15 min. The cells were blocked with 3 % goat serum and 0.1 % TritonX-100 in PBS for 1 h at room temperature, followed by the incubation with primary antibodies at 4 °C overnight. The second day, the cells were incubated with secondary antibodies after washed with PBS for 30 min. The images were taken with a Zeiss confocal microscope. Primary antibodies: Rabbit SOX2 (Millipore, Ab5603), Mouse Nestin (BD, #556309). Secondary antibodies: goat anti Rabbit 488 (Invitrogen, A11008), goat anti Mouse 568 (Invitrogen, A11004).

### 5hmC-specific enrichment and high-throughput sequencing

Chemical labeling-based 5hmC enrichment was described previously [26]. Briefly, DNA was sonicated to 100–500 bp, and then mixed with 100 μl solution containing 50 mM HEPES buffer (pH 7.9), 25 mM MgCl_2_, 250 μM UDP-6-N3-Glu, and 2.25 μM β-glucosyltransferase for 1 h at 37 °C. DNA substrates were purified via Qiagen DNA purification kit. 150 μM dibenzocyclooctyne modified biotin was then added to the purified DNA, and the labeling reaction was performed for 2 h at 37 °C. The biotin-labeled DNA was enriched by Streptavidin-coupled Dynabeads (Dynabeads® MyOne™ Streptavidin T1, Life Technologies) and purified.

5hmC libraries were generated with 25 ng input or 5hmC-captured DNA according to the manufacturer’s protocol (NEBNext ChIP-Seq Library Prep Reagent Set for Illumina). DNA fragments between 150 and 300 bp were gel-purified after the adapter ligation step. An Agilent 2100 BioAnalyzer was used to quantify the amplified DNA. 20 pM diluted libraries were eventually used for sequencing.

### Sequence alignment and mapped reads annotation

FASTQ sequence files were aligned to mouse NCBI37v1/mm9 references using Bowtie 0.12.9. Each unique mapped read with no more than two mismatches in the first 25 bp was concatenated to achieve combined wild-type and APP/PS1 mice 5hmC sequence. Association of mapped reads with genomic features was performed by overlapping reads files with known genomic features obtained from UCSC Tables for NCBI37v1/mm9. Unique 5hmC mapped reads were plotted to various genomic regions using an R program package termed ngsplot (https://code.google.com/p/ngsplot/).

### DhMR identification, annotation, and motif analysis

Model-based Analysis of ChIP-Seq (MACS) software [42] was adopted to identify DhMRs between WT and DTg mice by directly comparing one to the other, rather than comparing to the input. The effective genome size = 1.87 × 10^9^, tag size = 38, bandwidth = 200, *P*-value cutoff = 1.00 × 10^−5^. Identified WT and DTg-specific DhMRs were annotated to various genomic regions and associated genes by HOMER software [50]. DhMR-associated genes were extracted, and enrichment analysis was performed with WebGestalt (http://bioinfo.vanderbilt.edu/webgestalt/) [51].

### Statistics

Data are expressed as the mean ± standard error of the mean (s. e. m.), and statistical significance of differences between different groups was assessed using the *t*-test or ANOVA assay.

## Additional files

Additional file 1: Figure S1. A representative image of methylene blue staining showing the equal spotting of DNA in the membrane. Figure S2 5hmC chromosome-wide densities showing the distribution profiling on chromosomes. A depletion is observed on chr-X relative to autosomes. Figure S3 Representative IGV images show the decrease of 5hmC in some genomic regions of genes identified in two AD EWAS datasets. (PPTX 2495 kb) Additional file 2: Table S1. Reads info. (XLSX 10 kb) Additional file 3: Table S2. DhMR_DTg unique_annotated genes. (XLSX 10 kb) Additional file 4: Table S3. Aged DTg unique_annotated genes. (XLSX 39 kb)
