# Supplementary material for: Genome-wide alteration of 5-hydroxymenthylcytosine in a mouse model of Alzheimer’s disease
Source: BMC Genomics. 2016 May 20;17:381. doi: 10.1186/s12864-016-2731-1 (PMC4875608; doi:10.1186/s12864-016-2731-1)
Supplement: Additional file 1: Figure S1. — A representative image of methylene blue staining showing the equal spotting of DNA in the membrane. Figure S2 5hmC chromosome-wide densities showing the distribution profiling on chromosomes. A depletion is observed on chr-X relative to autosomes. Figure S3 Representative IGV images show the decrease of 5hmC in some genomic regions of genes identified in two AD EWAS datasets. (PPTX 2495 kb) [file 12864_2016_2731_MOESM1_ESM.pptx]

## Slide 1
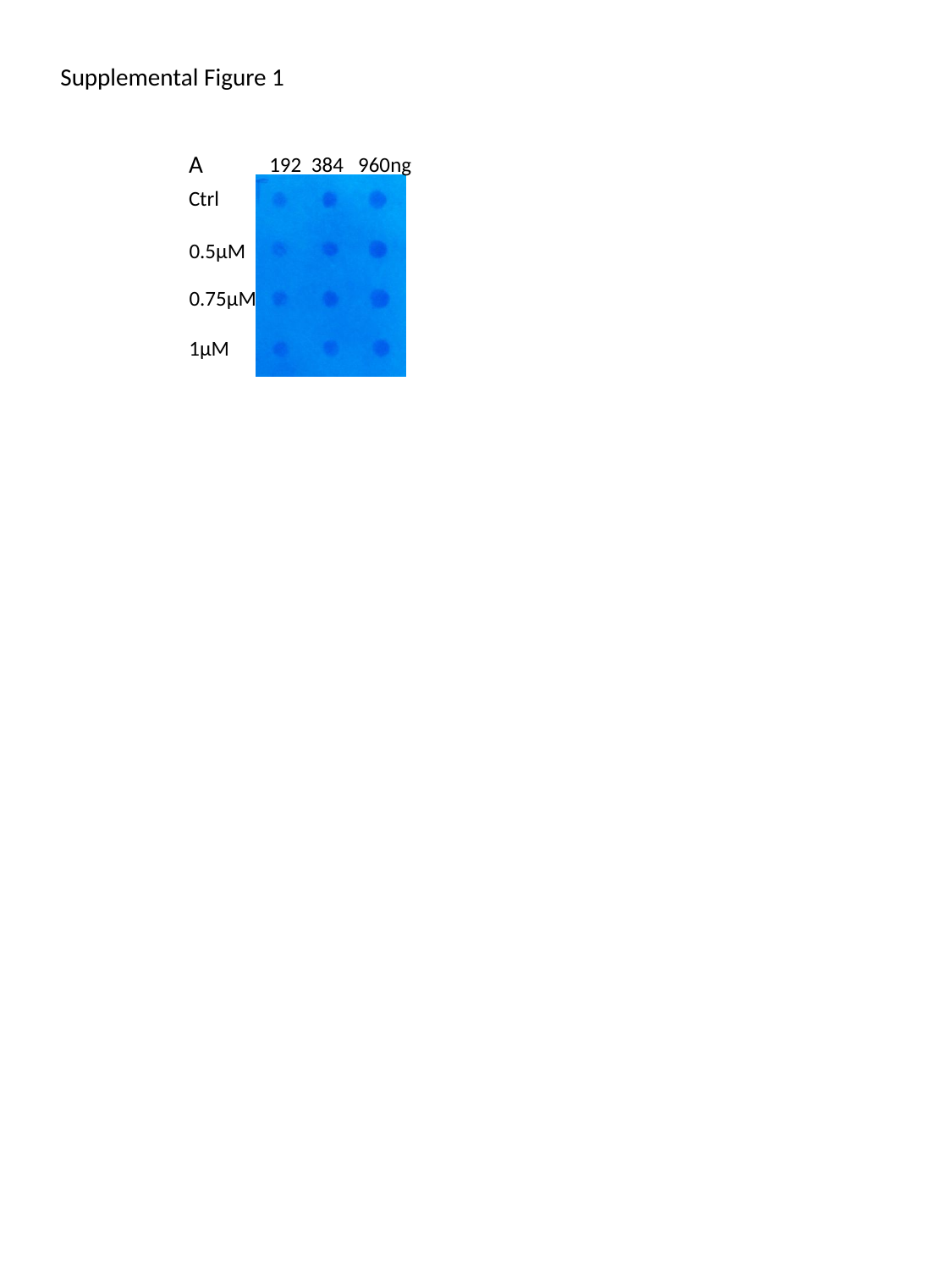

Supplemental Figure 1
A
192 384 960ng
Ctrl
0.5µM
0.75µM
1µM

## Slide 2
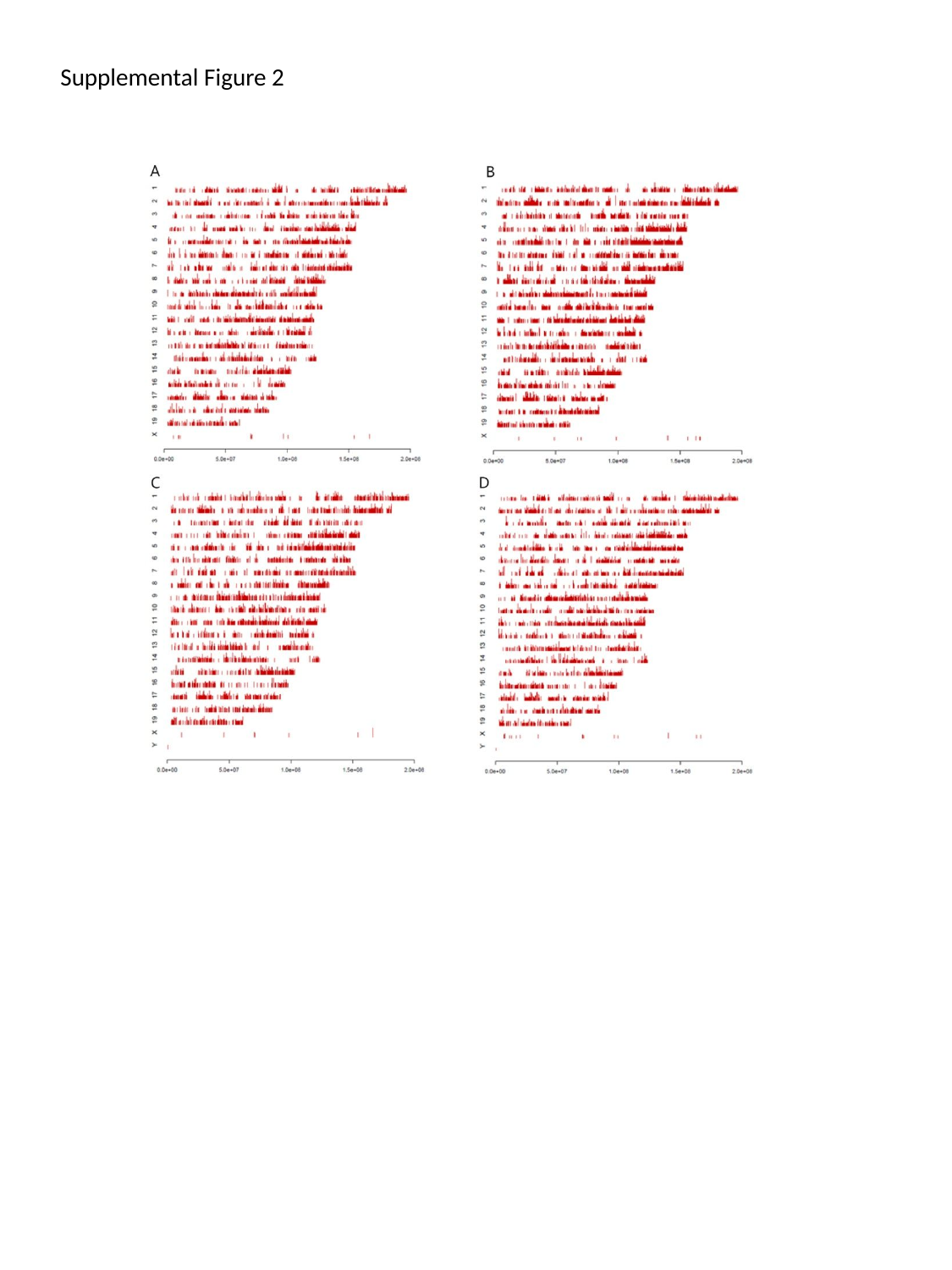

Supplemental Figure 2

## Slide 3
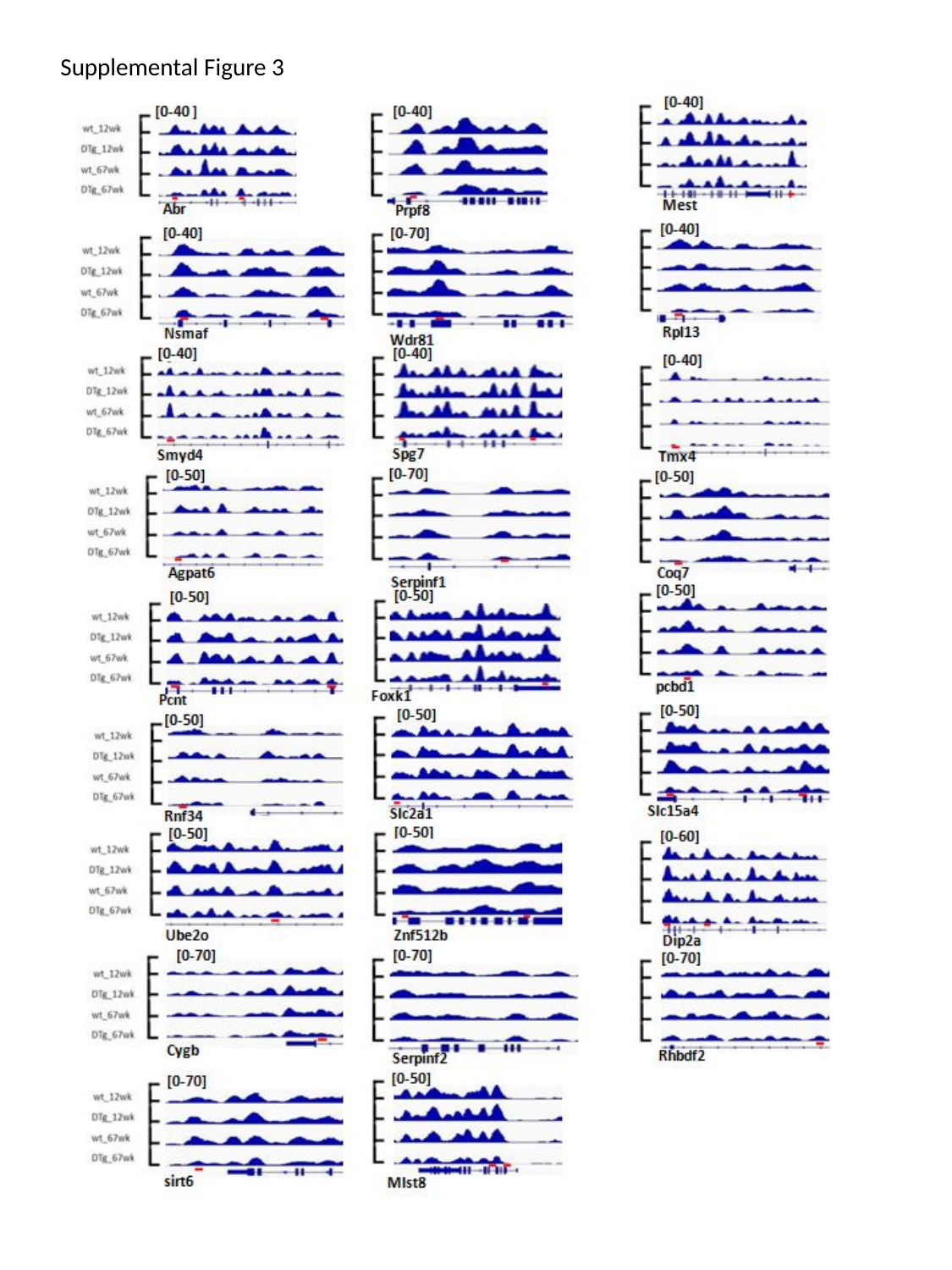

Supplemental Figure 3
